# Getting your DUCs in a row - standardising the representation of Digital Use Conditions

**DOI:** 10.1038/s41597-024-03280-6

**Published:** 2024-05-08

**Authors:** Francis Jeanson, Spencer J. Gibson, Pinar Alper, Alexander Bernier, J. Patrick Woolley, Daniel Mietchen, Andrzej Strug, Regina Becker, Pim Kamerling, Maria del Carmen Sanchez Gonzalez, Nancy Mah, Ann Novakowski, Mark D. Wilkinson, Oussama Mohammed Benhamed, Annalisa Landi, Georg Philip Krog, Heimo Müller, Umar Riaz, Colin Veal, Petr Holub, Esther van Enckevort, Anthony J. Brookes

**Affiliations:** 1 Centre for Analytics, Ontario Brain Institute, Toronto, Canada; 2 Genetics and Genome Biology, University of Leicester, Leicester, UK; 3 Luxembourg National Data Service, Esch-sur-Alzette, Luxembourg; 4 Faculty of Medicine and Health Sciences, McGill University, Montreal, Canada; 5 Nuffield Department of Population Health, University of Oxford, Oxford, UK; 6 Ronin Institute for Independent Scholarship, Montclair, USA; 7 Medical Laboratory Diagnostics Department, Medical University of Gdańsk, Gdańsk, Poland; 8 University of Luxembourg, Esch-sur-Alzette, Luxembourg; 9 Center for Radiology and Nuclear Medicine, VASCERN ERN /Radboud University Medical Center, Nijmegen, Netherlands; 10 Institute for Rare Diseases Research (IIER), Instituto de Salud Carlos III, Madrid, Spain; 11 Biomedical Data & Bioethics Group, Fraunhofer Institute for Biomedical Engineering, Sulzbach/Saar, Germany; 12 Governance Innovation, Sage Bionetworks, Seattle, USA; 13 Departamento de Biotecnología-Biología Vegetal, ETSI Agronómica, Alimentaria y de Biosistemas, Centro de Biotecnología y Genómica de Plantas (CBGP, UPM-INIA/CSIC), Universidad Politécnica de Madrid, Madrid, Spain; 14 Research, Fondazione per la Ricerca Farmacologica Gianni Benzi Onlus, Bari, Italy; 15 Legal, Signatu AS, Oslo, Norway; 16 BBMRI-ERIC, Graz, Austria; 17 Institute of Computer Science, Masaryk University, Brno, Czechia; 18 University of Groningen, Groningen, Netherlands; 19 Department of Genetics, University Medical Center Groningen, Groningen, Netherlands

**Keywords:** Standards, Software

## Abstract

Improving patient care and advancing scientific discovery requires responsible sharing of research data, healthcare records, biosamples, and biomedical resources that must also respect applicable use conditions. Defining a standard to structure and manage these use conditions is a complex and challenging task. This is exemplified by a near unlimited range of asset types, a high variability of applicable conditions, and differing applications at the individual or collective level. Furthermore, the specifics and granularity required are likely to vary depending on the ultimate contexts of use. All these factors confound alignment of institutional missions, funding objectives, regulatory and technical requirements to facilitate effective sharing. The presented work highlights the complexity and diversity of the problem, reviews the current state of the art, and emphasises the need for a flexible and adaptable approach. We propose Digital Use Conditions (DUC) as a framework that addresses these needs by leveraging existing standards, striking a balance between expressiveness versus ambiguity, and considering the breadth of applicable information with their context of use.

## Introduction

There is a widespread desire to maximise the sharing and reuse of research data, healthcare records, biosamples, and other biomedical artefacts. Sharing is often a requirement for funding. Yet, such activities must be conducted in a responsible manner that fully respects all the myriad ‘conditions of use’ that may apply. This is especially the case for sensitive and protected data and assets, including personal/healthcare information, commercially-valuable items, and products from competitive-domain endeavours.

Conditions of use for health data are defined at multiple levels of governance and through many different regulatory means. They may stem from ethical concerns, legal concerns, or even from a given institution’s mission, ethos, or funding. For instance, to reinforce statutory ethical and rights-based frameworks for data subjects in Europe and the UK, multiple legal documents and policy instruments such as the UK Data Act, the European Union’s General Data Protection Regulation (GDPR) and AI Act have been devised. Elsewhere, the revised Common Rule in the US and the Declaration of Taipei, an update of the World Medical Association’s (WMA) stance on the use of human subjects delineated in the Declaration of Helsinki, stipulate additional parameters for the uses of data. Law, soft law, and biomedical research ethics guidance like these are intended to help assure that data use and reuse to generate new discoveries will adhere to the applicable ethics standards and laws, and the rights of data subjects will be protected. These instruments help to engender public trust, and thereby ensure that valuable data will remain available for research and discovery. Yet, they also come at a significant cost. They can create a labyrinthine set of rules with no clear roadmap for detailing how they are to be implemented.

For this reason, there is a need for increasingly standardised ways to delineate, structure and manage the relevant conditions of use, suitable for use with previously created-assets and to support prospective activities. Defining a standard for conditions of asset use is, however, far from simple, primarily due to the immense diversity and scale of the challenge. First, there is a vast number of asset types that such a standard would need to relate to. Second, the applicable conditions of use can have many different origins, not least: individual subject consents; requirements stipulated by the asset’s owner/institution or the funding source that led to its generation; ethics committee procedures and decisions; and legal considerations that may be local, regional or international in nature. Third, the conditions may apply to discrete artefacts (e.g., single records, specific biosamples), to collections of such items (e.g., datasets from specific studies, clinical databases), or to whole resources (e.g., whole biobanks, institutional output). Fourth, the specifics and the granularity of the conditions of use are likely to differ depending on the specific use case, such as: formally documenting or communicating the conditions; informing the creation of support tools (consent forms, sharing contracts, etc); underpinning asset discovery services; or facilitating the automated triaging or processing of access requests. Clearly then, the challenge of data and asset sharing goes well beyond simply enabling resource custodians to express “access/sharing policies”, which itself is already a complex undertaking^1^.

The need to standardise ways to represent and leverage conditions of use continues to grow, coincident with the development of the internet, federated data technologies, and artificial intelligence (AI). Standardisation is an integral part of making datasets findable, accessible, interoperable, and reusable, or “FAIR”^2^. In tackling this challenge, one is torn between the desire to define a perfect and completely unambiguous semantic and syntactic model that would facilitate human and machine based understanding, and the pragmatic alternative of designing a flexible specification that would allow for some adaptations and ease of use in circumscribed contexts. There is also the question of the breadth of information that any such standard might attempt to cover. For example, one might define a relatively small ontology to merely support the exploitation of a limited range of dataset types based on headline conditions of use. Alternatively, one might define a complex semantic structure that would require a massive ontological underpinning and a sophisticated understanding of its design for appropriate use. Adding free text options into any approach would increase expressivity at the risk of adding ambiguity. Examples of all these approaches have been tested, and it is clear that no “one size fits all” solution yet exists or is likely to emerge. Instead, we propose there needs to be a series of solutions that tackle different aspects of the challenge where conditions of use representation is required.

In this report we introduce the Digital Use Conditions (DUC) as a framework that balances and establishes a consistent community platform for addressing these needs. The purpose of DUC is to provide end users with syntactically consistent solutions to the various inconsistencies that arise when multiple languages and ontologies for use conditions are employed across institutions and regions. This consistency makes the communication of use conditions more efficient. The increased efficiency can support more effective coordination among data producers, data users, and data oversight bodies as they navigate the many technical, ethical, and regulatory intricacies surrounding their work.

Previously, in 2016, Dyke *et al*. proposed a set of 19 arbitrary codes that sought to capture an overview of permissions for secondary use of genomics datasets in research and clinical settings^3,4^. These “Consent Codes” comprised an unstructured set of labels separated into primary categories, secondary categories, and requirements. While datasets should fall under a single primary category, additional secondary categories and requirement codes could be applied to refine conditions of use. The model provided no way to vary, elaborate or reverse the meaning of any of the coded terms. Nevertheless, since it was based on concepts of use that were commonly employed, Consent Codes were a valuable starting point for establishing some automatable structure within this domain.

Subsequently, the Consent Code terms were used as a basis for the formal “Data Use Ontology” (DUO)^5^ generated by the Global Alliance for Genomics and Health (GA4GH). As part of this work, the term definitions were made more precise and additional terms were added. DUO further separated terms into ‘permission terms’ and ‘modifier terms’ to be combined. Permission terms generally stipulated the type of research to be allowed (e.g., population level versus disease specific level research), whilst the modifiers added specific limitations/prohibitions to those categories of use (e.g., ‘Collaboration required’, ‘No general methods use’, ‘Genetic studies only’). DUO is increasingly used in practical settings to encode common conditions of use, especially relevant to genomics research data. For instance, EBI, BBMRI and the NIH have implemented DUO in key repositories to facilitate the discovery of datasets or samplesets based on usage terms [e.g., BBMRI-ERIC Directory^6^, or European Genome-Phenome Archive^7^].

Other important efforts in data use ontology modelling include the Informed Consent Ontology (ICO)^8^ and the Agreements ontology (AGR-O)^9^. While ICO offers an expansive set of terms related to consent terminology, AGR-O follows a more granular approach than the DUO and ICO vocabularies. However, a granular representation of Data Use Agreements and Data Use Limitations distinguishing permissions, prohibitions, and obligations can further increase the difficulty of the task. This can frequently become intractable because the original governance documents did not consider such detailed descriptions and so selecting relevant terms can become difficult.

To extend the flexibility and utility of the Consent Codes and DUO approaches, Woolley *et al*. devised the “Automatable Discovery and Access Matrix” (ADA-M)^10^. This provides a data structure to hold an extended set of 42 optional conditions of use terms, which when entered into that structure constitute an ADA-M “Profile”. Uniquely, this design: (a) ensured that each term was purely ‘atomic’ (i.e., unlike its predecessors, each term never conflated more than one concept of use); and (b) eliminated ‘directionality’ from all the terms (i.e., definitions were silent on whether the concept of use was allowed or not allowed). These ‘pure’ concept of use terms were employed with adapters whereby each modality of use could be given a directionality (as “Unrestricted”, “Unrestricted[Obligatory]”, “Limited”, “Limited[Obligatory]” or “Forbidden”), whilst terms that referred to a conditionality were declared as “True” or “False”. Header and Meta-Condition sections were also provided to contextualise the ADA-M Profile. Critically, the Header enables ADA-M to provide useful capabilities not afforded by Consent Codes or DUO. Specifically, codes and ontology based systems typically function as ‘tags’ to be appended onto datasets, whereas an ADA-M Profile can similarly be appended or it can act as a self-standing statement of use conditions, with an optional internal pointer (in the Header) to reference whatever asset(s) it pertains to. This increases the ways in which conditions of use can be assigned.

Attempts to achieve flexible and expressive mechanisms for conditions of use based upon the W3C semantic web resource description framework (RDF) have been underway in the broader digital information community. The Open Digital Rights Language (ODRL)^11^ model endeavours to provide a comprehensive solution built upon stating a set of rules and relationships between ‘assets’, ‘policies’, ‘duties’, ‘constraints’, ‘permissions’, and ‘prohibitions’. Other related efforts include the Open Data Rights Statement vocabulary (ODRS)^12^ which is focussed on representing digital licences. The Data Tags Suite (DATS)^13^ model was designed to meet distinct objectives aimed at formally expressing conditions for asset use in the life sciences. Complexity, however, can represent a significant dis-incentive for groups without semantic web expertise. It is therefore daunting to imagine how one might design a semantic or syntactic standard that could support any and all sophisticated conditions of use applications, whilst still remaining possible to use correctly and not imposing an extreme burden of adoption.

Standardised ontologies and vocabularies (such as Consent Codes, DUO, ICO, and others) act as standalone metadata “tags” or “labels,” that follow data throughout their life, and provide simplified representations of the full permissions associated to a dataset. This is especially useful for prospective efforts to assign common permissions to newly-generated data that can interoperate with other data. The advantages include user-friendliness, low barriers to adoption, and compatibility with automated systems that strive toward the discovery of datasets that are subject to compatible or harmonised data governance rules. They can also help design data governance rules in streamlined formats according to shared methodologies. However, for pre-existing or other datasets that are subject to heterogeneous or non-interoperable conditions of data use, it might prove impracticable or impossible to use ontologies to accurately capture this information. Conversely, representation systems that provide both semantic terms but also a syntactic structure to define more complex conditions, such as ADA-M, can enable the full range of governance rules applicable to a dataset to be captured, even if such datasets are subject to complicated or unique data governance rules. This makes them particularly applicable to pre-existing, retrospective data that are subject to complex governance rules. Standardised ontologies have low implementation costs but require significant pre-implementation work to ensure that the governance rules applicable to the concerned data are relatively compatible. However, more complex systems such as ADA-M, ODRL and others have higher barriers to adoption for organisations, in the form of both training and labour required.

Given the state of the art in recent years, and the remaining need for better standardised ways to express and structure conditions of use, a group of over 40 scientists, technicians, and other stakeholders worldwide began collaborating in 2020 to identify areas of unmet need and propose solutions. The group was constituted as a Task Force of the International Rare Disease Research Consortium (IRDiRC), and worked with the teams from the European Joint Program for Rare Disease (EJP-RD) project to undertake alpha-testing of specifications, tools and vocabularies as they progressively matured. This resulted in the new ‘Digital Use Conditions’ specification as described here, for structuring conditions of use, which is designed to be elegantly simple to use, and yet flexible in scope and applicability by virtue of being able to employ any set of use condition concepts as an underpinning semantic layer. It effectively leverages existing semantic vocabularies like DUO and ICO while adhering to atomicity, it provides the useful modularity of ADA-M without its complexity, and affords the creation of intuitive yet flexible sentence-like conditions with user defined or semantic web compatible terms.

## Results

**Fig. 1:**
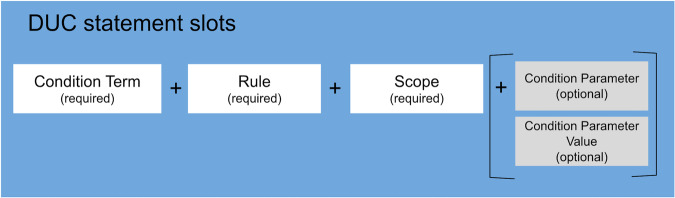
Main facets of a DUC profile that form a simple yet flexible structure for describing digital use conditions of health and research information assets.

The DUC model is proposed as a syntactic informational standard for representing conditions of use metadata, along with optional contextual data. The full specification is accessible via 10.5281/zenodo.7767323. The semantic terms, concepts and definitions that would be used in conjunction with this syntactic model are purposely left undefined so that it can be used flexibly with whatever application ontologies or standard ontologies that are most suited to the area of interest. The core DUC model is shown in Fig. 1, and was conceived with various key objectives and principles in mind.

First, the proposed DUC structure was designed so that it should in principle be able to represent conditions of use information for any type of scale of biomedical resource or object. This might include individual data records, individual biosamples, collections of records or samples, or whole biobanks and data stores. For convenience, we refer to all such possibilities as ‘assets’. It would not be practical to demonstrate compatibility with all possible assets in a first report of the DUC structure, and so we settled on validating the use of DUC in the context of whole biobanks and patient registries.

Second, the model should be equally applicable regardless of whether the default operational assumption is that all forms of asset use are permitted unless explicitly ruled out, all uses are not allowed unless explicitly granted, or where no default assumption exists. A dedicated “*permissionMode*” attribute in the DUC model (see below) allows one the option of specifying which default, if any, applies. Directly related to this, DUC adopts the approach of ADA-M whereby the underlying concepts of use (from whatever ontology may be employed) must be non-directional, with directionality being asserted for each referenced concept as part of the creation of a DUC ‘Profile’ (i.e., populated instance of the DUC model).

Third, when multiple conditions of use statements are composed into a DUC Profile, these should not be taken to have any explicit or implicit inter-dependencies. This is a strong design decision, which is recognised to limit the expressivity of the DUC model, as sometimes such inter-dependencies will exist. Several options were considered for conveying Boolean logic that could exist between conditions of use statements, but when tested in practice this added level of complexity caused considerable confusion amongst adopters, and the resulting flexibility meant that sharing/access policies could unhelpfully be formulated as different but equivalent Profiles. Neither of these situations was deemed attractive for a first version of the DUC model. Instead, the aim was to keep the initial design clean, consistent, and intuitive to promote widespread adoption. We anticipate that later versions may be elaborated and optimised to support more nuanced and granular conditions of use arrangements.

Fourth, the DUC structure should in principle bring a degree of utility for any and all mainstream use cases. This includes capture, documenting, representing and communicating primary conditions of use of an asset, guidance for governance tool generation (e.g., forms, contracts, software), support for asset discovery services, and support for automated triaging and decision making to assist the work of Data Access Committees. This range of use cases ultimately boils down to whether or not conditions of use can be represented in a consistent and unambiguous manner. Achieving this sufficiently to enable unsupervised, perfect machine interpretability is unrealistic, and so this design principle is really about seeking to achieve a useful degree of functionality. Our development and testing of the DUC structure has explored this for all above use cases, other than automated triaging.

The core of the DUC model comprises a structure by which one or more conditions of use statements can be asserted. Each statement comprises three required parts, namely:

- A required “*conditionTerm*”, which is the atomic and non-directional concept of use, which may be entered as free text (in the “*conditionTerm.label*” sub-field) but ideally would be defined by a term from a standard ontology, a documented application ontology, or a controlled vocabulary (in the “*conditionTerm.uri*” sub-field). There should be a limited number of such concepts used in any setting, each designed to be as general as possible, to match the domain of application. This way, at the level of the *conditionTerm* the statements will be very straightforward and unambiguous.
- A required “*rule*” which determines the directionality of the *conditionTerm*, for which acceptable values are “Obligatory”, “Permitted”, “Forbidden”, and “No Requirement”.
- A required “*scope*” field which establishes whether the *conditionTerm + rule* combination applies to the “Whole of asset” or only “Part of asset”. The default would be “Whole of asset” except in the case of some multi-element type assets (for example, not all samples in a biosample collection may be approved for use in profit-based research)

This core structure provides a simple and yet flexible and consistent way to represent basic conditions of use, but in many cases there will be a need for more precision. To facilitate this in a manner that retains the model’s simplicity and yet facilitates as much computer-readability as possible, a fourth and optional section is provided for each statement, namely:

- An optional “*conditionParameter*” field, by which each statement can be made more detailed and precise, to any degree desired. The *conditionParameter* content should not refer to other statements in the Profile, as each is an independent assertion. The *conditionParameter* can include free text (via the “*conditionParameter.label”* sub-field) or reference an ontology term such as a country code or disease name (via the “*conditionParameter.uri*” sub-field) to bring a greater degree of computer readability. In situations where a specific value would be useful to state, this is facilitated by using the “*conditionParameter.value”* sub-field, e.g., “2” if data destruction is required after a certain number of years. Despite providing this optional sub-structure for *conditionParameter* content, the DUC design deliberately also offers the free text alternative, to promote adoption and easy use of the DUC model. Subsequent versions may refine this section, based on feedback from its use by the community.

When formulating a condition statement based upon the above, it is essential that the elements are assembled in the given order and following a very specific logic: First, one starts with a “conditionTerm” root which is atomic and non-directional. Second, adding the “rule” converts this into a directional but still atomic and meaningful concept of use. This 2-part statement might sometimes represent a term in an existing ontology, and so may be conveniently equated as such. Third, one adds the “scope” element, which must specifically refer to the ambit or coverage of the preceding 2-part statement. For example, for the directional concept of use statement created as {‘Use for profit purposes’ (“conditionTerm”) is ‘Permitted’ (“rule”)} the logical “scope” might be ‘Whole of asset’ if all samples in a biocollection were permitted to be so used, or instead would be ‘Part of asset’ if this depended upon some other consideration (such as individual consent or remaining sample volume). Fourth, the “conditionParameter” is then optionally appended if one wishes to elaborate/explain the preceding 3-part statement. For example, as per the previous example, one might want to indicate the dependence upon individual consent, or in another context one might want to add one or more country codes to elaborate the 3-part statement {‘Use in a geographic region’ (“conditionTerm”) is ‘Permitted’ (“rule”) for ‘Whole of asset’ (“scope”)}.

When combined, the four parts of each statement are intended to be intuitive, in that they together provide a sort of natural sentence, as follows: “Regarding [*conditionTerm*], this form of use is [*rule*], and applies to the [*scope*], for which the details are [*conditionParameter*]”.

**Fig. 2:**
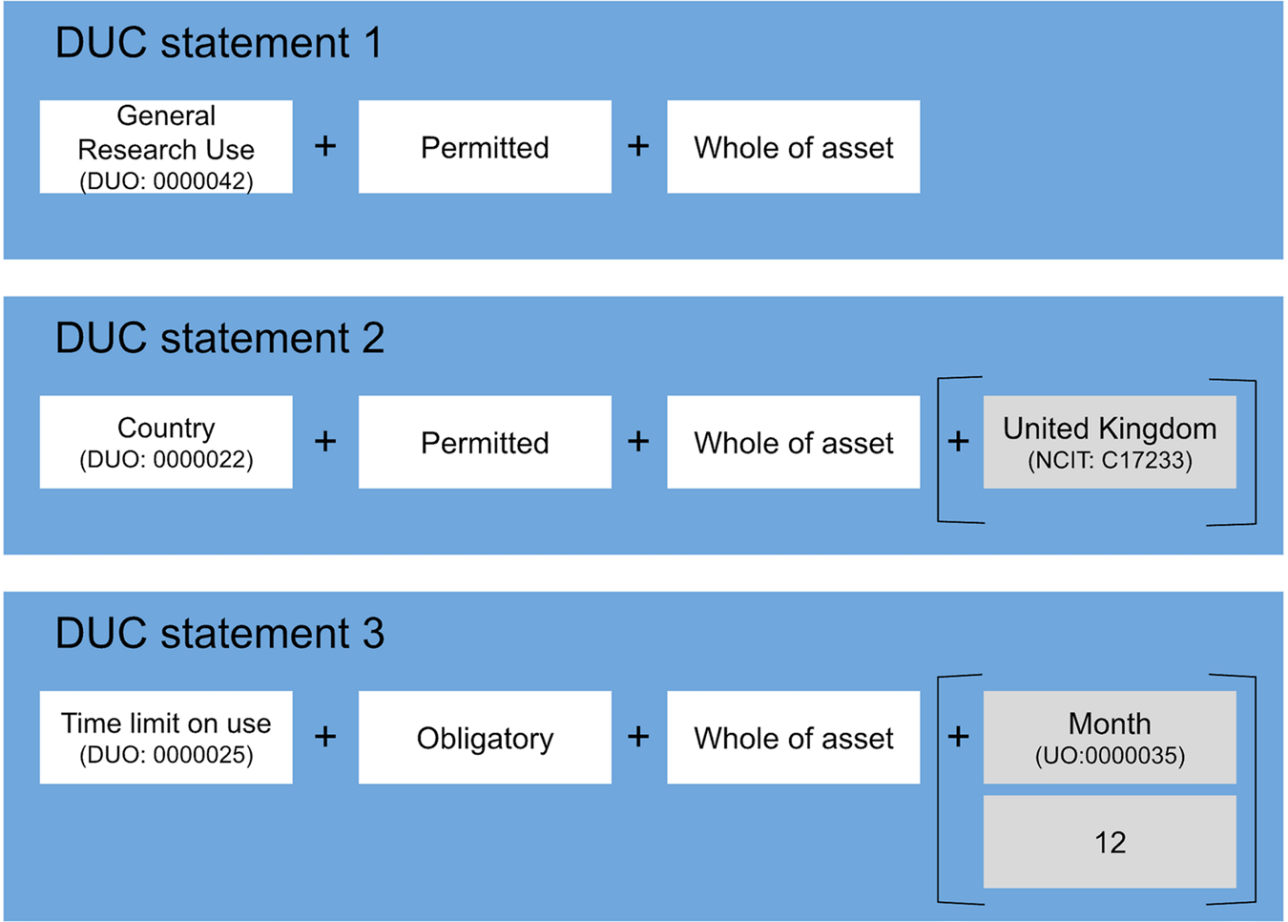
An example of a DUC profile consisting of 3 DUC statements.

By way of example, Fig. 2. illustrates conditions of use statements that could be placed into a single DUC Profile formed with this core DUC design.

DUC Profiles combine multiple (at least one) independent statements of equal standing, as per the example in Fig. 2. This example states, “General research use is permitted for the whole of asset. The country of the United Kingdom is permitted for the whole of asset. The time limit on use is 12 months and is obligatory for the whole of asset.” Extending this approach, one could create multiple Profiles for a single asset in order to indirectly represent inter-statement relationships – for example, Profile-1 might state that asset use is permitted within countries A, B and C and also for profit-based research, whereas a simultaneously applicable Profile-2 might state that asset use is permitted in countries D, E and F with profit-based research not being allowed.

**Fig. 3:**
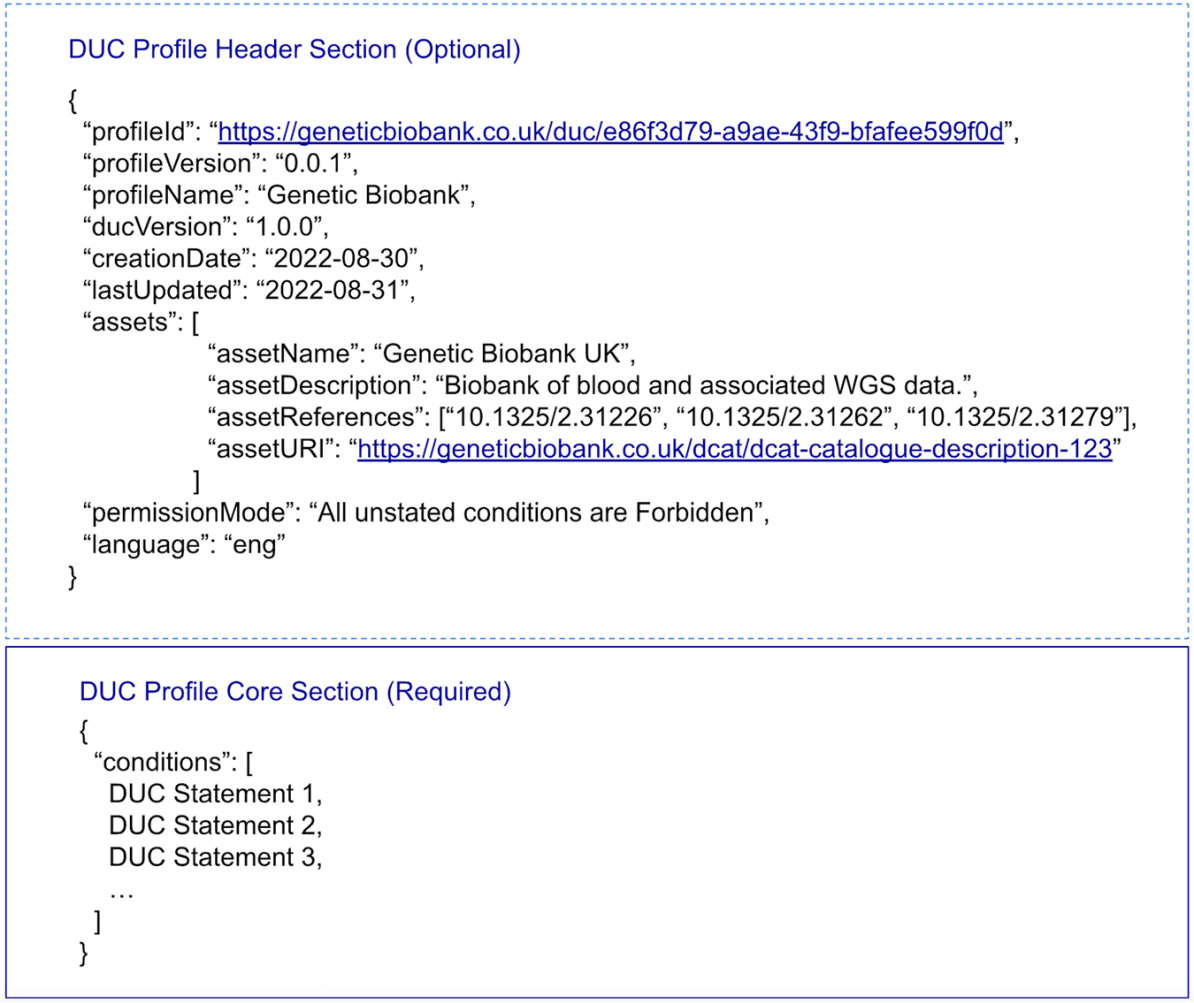
Fictional example DUC Profile, using optional contextualisation fields. The DUC header provides the contextual fields for the 3 DUC statements in the core (detailed in Fig. 2).

Beyond the multi-statement core of a DUC Profile, the model also offers a number of other fields to contextualise the conditions of use statements, provide administrative guidance, and reference the asset(s) to which the profile applies. All of these fields are optional, as in some cases a profile will simply need to comprise the core conditions of use statements to act as an informational object that is pointed to by an asset. These additional fields are as listed below, and an example of their use is provided in Fig. 3.:

- “*profileId*” a unique profile ID resource identifier (URI) that uniquely identifies the profile in a way that makes the DUC profile findable and identifiable. Ideally, this would be a publicly web accessible URI. We recommend the use of a universal unique identifier (UUID) as part of the URI in order to avoid ambiguous profile identifiers.
- “*profileVersion*” a semantic version of the DUC Profile (e.g., 1.0.1) that enables the creation of multiple versions of a profile in case changes to the terms evolve over time, but where prior terms must be archived or honoured in the context of agreements.
- “*profileName*” a human readable string providing a name for the profile.
- “*ducVersion*” the version of the DUC schema utilised by the profile.
- “*creationDate*” a date object using the ISO 8601 standard to capture the date the DUC Profile was first created.
- “*lastUpdated*” a date object using the ISO 8601 standard to capture the date the DUC Profile was last updated.
- “assets” which specifies an array of one or more assets that the DUC Profile applies to. This option of having the DUC Profile point to its referenced assets will sometimes be needed, but a more intuitive strategy would be to have the metadata of those assets point to the relevant DUC Profile(s) that apply, or to have assets and their DUC jointly referenced by some cataloguing service. Each asset listed by this array can be described by several subfields, namely:
  - “*assetName*” a string to capture the name of the asset.
  - “*assetDescription*” a string to describe the asset.
  - “*assetReferences*” an array of strings to capture web links or names of publications and other references that describe the asset.
  - “*assetURI*” a URI to point to an online object that formally defines the asset in question.

- “*permissionMode*” a field to choose between “All unstated conditions are Forbidden” and “All unstated conditions are Permitted”, to explicitly declare how unstated conditions should be interpreted.
- “*language*” an ISO 639-3 three letter code defining the language used in the DUC profile.

## Discussion

The DUC model described above resulted from over a year of iterative testing and refinement of the design. This work entailed over 13 groups involved in biobanking and rare disease patient registry construction, and tapped into their experience of what would be the main conditions of use concepts to cover, what granularity was needed to serve the documenting and discovery use cases, and what level of design complexity would match the ability of users that would populate and consume DUC Profiles.

This very practical approach to standard development ensured that the model struck a balance between being sufficiently powerful to be useful and yet convenient and intuitive enough to be usable by typical adopters. Key decisions that came out of this included:

- The principle of leaving the semantic layer completely open, so that this dimension can adapt to specific domains and use cases, and become increasingly standardised with time.
- The choice and number of non-core (contextualisation) fields.
- The notion that all contextualisation fields should remain optional.
- Allow free text values for some fields rather than trying to tie everything rigidly to formal ontologies and complex substructures.

The aim was to devise a syntactic model that affords more utility than previous ontologies or the ADA-M specification, whilst not seeking to create an ultimate solution that would support all possible governance-related use cases with 100% precision and machine readability. As DUC becomes used in practice, we anticipate further evolution of its design based on practical experience and resulting feedback. Indeed, a number of major programs have already signalled their intention to adopt DUC, and work towards future improvements and specialisations. For instance, the EU project EJPRD (a data infrastructure for rare disease) and the IHI EPND project (a data platform for neurodegenerative disease) have adopted DUC and are currently building it into their systems.

The IRDiRC Task Force developed DUC specifically to establish a method and structure for clear communication in regulatory contexts where there is currently very little communicative clarity with respect to the conditions of use for digital assets. The various conditions of use which must be respected are defined and delineated at multiple levels of governance and through many different regulatory means. They may stem from long standing ethical principles, new legislation, or from a given institution’s mission, ethos, or funding. Various stipulations originating from these multiple origins do not easily combine into an efficient, or even functional, system for day-to-day data governance.

There may often be a degree of consistency across these multiple levels. For example, in the case of personal biomedical data, one of the most highly regulated data types, regulatory requirements for creation, discovery, and access of data are often delineated in ways reflective of longstanding and widely accepted bioethical principles. In many cases, they are also to be managed in accordance with rights-based legal frameworks, such as human rights. Many institutional best practices, codes of conduct, and mission statements also draw from these same ethical and legal cornerstones.

However, in practice, the practicalities of implementing use conditions can vary across jurisdictions and institutions. Sometimes these vary in their objectives. And sometimes, even where objectives align, they vary in the specific language and in the modes of expression they employ. Even in cases where different jurisdictions all share the same principal legislation, for instance the GDPR, interpretation and implementation of the legislation is intended to be flexible, responsive to different social, cultural, and linguistic variables across regions. In many areas of the world, no such baseline legislation even exists. The US, for instance, has no primary data protection regulation. Instead, there are scores of different laws, both federal and state, which must be variously applied as needed, depending upon the state, the contexts, and the circumstances.

Typically, responsibility for preserving the ethical and legal legitimacy of data management practices falls to institutionally or statutorily required oversight bodies, such as research ethics committees (RECs), institutional review boards (IRBs), data access committees (DACs), or various data controllers. It is the responsibility of these bodies to either approve or deny requests to access data under their purview. In the past two decades, these bodies have faced quite formidable challenges. At a time when the sheer number of data access requests are already severely taxing their resources, they may find themselves in an unmanageable situation. Frequently, they are mandated to make data accessible while, at the same time, they are also mandated to enforce a host of legal and ethical parameters which limit access. These two opposing duties are not necessarily always consistent or easily harmonizable. Furthermore, these duties must be carried out while also demonstrating adherence to an institution’s own mission statement, ethos, and code of conduct. The result leads to costly, time consuming, and labour-intensive work.

To make data management practises more cost effective and efficient, various ontologies for data use conditions have been created to enable automation of certain processes. Yet, for many potential end-users, the semantics and syntax these ontologies employ are often not sufficiently descriptive or fit for purpose beyond a certain scope. As a result, different ontologies are selected by different institutions. These variations allow semantic and syntactical diversity to arise which prevents there being a clear and consistent means for communicating use conditions from one institution to another. These differences become barriers to establishing widespread interoperability. The lack of consistency makes it difficult for researchers whose projects require access to data from multiple institutions or across jurisdictions to communicate effectively with the respective oversight bodies about how ethical and regulatory matters may or may not pertain to the proposed research. In the end, it is simply left to data producers, data users, data managers, and data oversight bodies to judge how to muddle through this rather dysfunctional regulatory environment.

DUC was designed to help its end users address these kinds of communicative challenges for managing data and other assets. The purpose of DUC is to create the means for efficient access to data and assets by enabling end users who are not experts in the data sciences to easily produce a meaningful and accurate representation of use conditions, while minimising problems that arise from the many linguistic and semantic complications discussed above. The simple and straightforward strategy DUC employs means that end users can do this without having to undergo hours of training or rely on complex technical manuals on the many idiosyncrasies of a given ontology or data management system. This will enable end users to progress their research, while still demonstrating that institutional missions, codes of conduct, and regulatory matters are being attended to.

Even in this first version, DUC has been designed to support quite a wide range of asset types and use cases. The most obvious one is the capture and documentation of principal conditions of use for collective assets such as biobanks, databases, registries, image collections etc. This is where we have focused our validation efforts so far, but work has also been initiated to explore support for discovery services, guidance for tool/form/contract development, and mapping to advanced semantic web models. Initial findings suggest that DUC does offer considerable utility in these areas as well.

From the outset, we aimed to ensure that DUC would be interoperable with existing controlled vocabularies and ontologies. As described, the core DUC model makes use of “label” and “uri” attributes for both the conditionTerm and conditionParameter properties of a single condition. Label attributes allow the entry of any free-text elaboration of the conditionTerm or conditionParameter, for example {conditionTerm.label: “Disease specific research”}, or {conditionParameter.label: “Epilepsy”}. While labels allow for maximum flexibility, where one may choose to either use an existing controlled vocabulary term or a concept from their own design, this approach may lead to greater variability and low interoperability. If a data user is seeking to access all data permitted for epilepsy research, but one data custodian labels their conditionTerm.label as “Research specific” while another custodian labels their conditionTerm.label as “Disease specific research”, the query system will either fail to resolve that both sources are available, or will require an intermediary capable of discerning when two labels *mean* the same thing or not. To reduce the likelihood of this from occurring, the uri attribute can be used to make use of the rich and rapidly expanding ecosystem of controlled terminology that are referenceable via persistent urls. For example, one can make use of the DUO code “DUO:0000007” corresponding to “Disease specific research” and available via persistent url at http://purl.obolibrary.org/obo/DUO_0000007. Similarly, the uri attribute for conditionParameter can make use of existing ontologies such as the Human Disease Ontology (DOID) to refer to the concept of “epilepsy” with the persistent url: http://purl.obolibrary.org/obo/DOID_1826. As a result, a more formal and machine readable definition can be created for the example above by making use of uri attributes. For example, one could construct the above DUC condition as {conditionTerm.label: “Disease specific research”, conditionTerm.uri: “http://purl.obolibrary.org/obo/DUO_0000007”, conditionParameter.label: “epilepsy”, conditionParameter.uri: “http://purl.obolibrary.org/obo/DOID_1826”, rule: “Permitted”, scope: “Whole of asset”}. It is important to note that the uri attribute does not require the use of http based urls but can in fact refer to any unique resource identifier that may or may not include a locator protocol such as http. Despite the relatively large set of controlled vocabularies and ontologies to choose from, substantial efforts in the community have led to formal mechanisms for matching between controlled terms, which suggests that DUC condition ambiguities will be much more readily resolvable using the uri attribute rather than the free-text label attribute for conditionTerm and conditionParameter.

Interestingly, by our testing of DUC it became apparent that all conditions of use statements can be classified as “who”, “what”, “when”, “where”, “why” and “how” forms or requirements of use. We then realised that some of the permitted *rule* options may not be especially useful or even very logical when paired with some of these categories (for example a *conditionTerm* for a “how” concept such as “ethical approval” makes little sense with the “forbidden” *rule*), but in the design of DUC it was agreed that no such combinations should be disallowed. Adopters might, however, choose to create tools and interfaces for DUC profiles creation that could act to impose such limitations to further ease their creation.

Three areas where we anticipate further development of DUC might be prioritised include: (i) providing a mechanism whereby Boolean relationships and conditionalities between conditions of use statements can be specified - this could help remove the need to create separate DUC profiles for distinct sets of terms as discussed above; (ii) a more structured and sophisticated design for the *conditionParameter* portion of each conditions of use statement in order to support scenarios such as formally coding time-spans and other more complex parameter values; and (iii) further explore the alignment of DUC with existing rights expression languages, such as ODRL. In each case some work on this has been undertaken, but it quickly became obvious that the added complexity imposed major challenges to ease of adoption. Wider practical use and feedback on DUC therefore becomes a prerequisite in guiding these areas of future development.

Another area of further development relates to tailoring the DUC design to directly support the capture and management of patient-specific consent. The IRDiRC Task Force that has devised the current version of DUC will now work to explore meeting this area of need. It will build on a consent form design created for the rare disease community, and explore DUC Profile storage options that would support dynamic consent environments.

While we believe the heightened flexibility of DUC Profiles is a benefit overall, there remains the potential for organisations to implement DUC Profiles in an incompatible manner. For example this can occur if different ontologies are used, or if free text fields for conditions and their details are used in a liberal way without pointing to formal ontologies. To address this, we have begun experimenting with artificial intelligence (AI) large language models (LLMs) to evaluate the feasibility of creating tools that would automatically convert sets of institutional contracts and consent terms into DUC Profiles, as well as converting DUC Profiles into human friendly natural language summaries. Another potential challenge for DUC adoption is the possibly higher implementation costs in terms of time and technology. Implementers will be required to serve dedicated files or API endpoints for DUC Profiles. We believe, however, that this structured model provides a simple yet powerful syntactic structure to be combined with existing ontologies to produce simple to granular human or machine readable use condition terms.

To facilitate and expand the adoption of a health data use permission structure such as DUC, it will be important for our working group to engage with the international research community even more broadly. In particular, major consortia hosting health data available for research should be engaged as well as major research centres looking for a solution to facilitate greater access to health research data. By expanding participation, an even more equitable and capable model for DUC can be evolved over time, in particular in its capacity to interoperate with additional ontologies, vocabularies, and nomenclatures for richer semantics, as well as technical systems integrations such as application programming interfaces (APIs) for accessibility and use.

In summary, the DUC syntactic model has been devised as an attempt to bring together many features and advantages of previous standard developments in this space, with the aim of providing enhanced utility and flexibility without imposing excess complexity and associated challenges to adoption. The IRDiRC task force behind this initiative would welcome more members to the group, and/or would encourage efforts by others to take DUC forward in new and exciting directions.

## Methods

In 2020, an international group began discussing current limitations with data models aimed at representing conditions of data access and use within and outside the context of health. Consulting with IRDiRC and reaching out to additional stakeholders, a task force with over 40 members was formed. Regular meetings were organised beginning in the fall of 2020 and continue to this day. This group remains open for other experts to join. As a first step to identifying the limitations with the current models, an extensive review of existing standards and methods for expressing conditions for access and use of health data was conducted. Many members, who are also authors of this manuscript, had already made significant contributions in this area. In time, a general consensus formed that – while versatile expression languages such as ADA-M and ODRL existed and controlled vocabularies such as Consent Codes, DUO, and others were available – no simple yet sufficiently expressive structure yet existed which could extend vocabularies formally while also maintaining a simplicity in design and use suitable for adoption by non-experts.

To initiate the creation of a data model design, suitable for experts and nonexperts alike, we first considered a kind of minimal syntax for expressing simple statements akin to a natural language syntax. Statements in many natural languages can be constructed simply with a noun phrase followed by a preposition phrase and a verb phrase. These three components alone can create syntactic connections that define rules which establish necessary conditions for semantic relations. The combination of syntax and semantics together define parameters for coherent meaning, in essence, rules for a meaningful statement. In health data sharing contexts, this constitutes the rules for use, in other words, Data Use Conditions (DUC). For example, we can adopt this approach to construct meaningful conditions of use statements such as “research use in epilepsy is obligatory” or “time limit of use of 12 months is permitted”. Surprisingly robust and expressive, the semantic structure of such simple statements led us to its adoption for the core DUC conditions structure.

By combining many such statements, a “profile” for use conditions pertaining to a given dataset or other asset can be formed. Efforts for expressing more complex statements are still underway, however we opted to keep a simple structure for the first version of DUC because identifying the simplest syntactical foundations to all data use conditions is a necessary first step in designing interoperable tools. Subsequent testing has borne this out and shown that most sets of data use conditions, expressed in terms of any number of semantic variants, can be shown to be syntactically well-defined with the help of one or more “DUC Profiles”.

After determining which fields should be mandatory and which should be optional, we worked to refine the labels used for each field as well as for fields that required values to be defined. In particular, rule option values were adopted with contribution from legal experts while condition term keys and optional fields were adopted with consideration for various health data repositories. Testing was undertaken during this design process. In particular, the use of existing ontologies such as DUO terms were tested. After DUC specification terms were defined, we adopted the JSON Schema 2020-12 specification to define DUC version 1.0.0^14^.

To support this work, online tools and code were created for DUC Profile construction (available at 10.5281/zenodo.7767323) along with web links to instructional help manuals and guides that were written to address questions and areas of confusion as they were identified. Wherever possible, we based *conditionTerm* options upon conditions of use concepts from existing ontologies, or devised application ontology terms when recommended by the system testers. While existing ontologies and other controlled vocabularies could satisfy many condition scenarios, a number of Taskforce members identified a gap in availability of simple and unambiguous atomic terms to meet common health data use scenarios. As a result, further details around semantic specifications that could support DUC are being developed. This includes ‘Common Conditions of use Elements’ (CCE) which comprises a set of atomic concepts designed within the European Joint Programme on Rare Diseases (EJP-RD) which, with extensive testing, are proving to work particularly well with the DUC structure^15^.

## Data Availability

No data were generated for this work, instead a JSON schema specification was developed and is made available as described in the Code Availability section.

## Code availability

The DUC specification is available as a JSON schema accessible via the following URL: 10.5281/zenodo.7767323. This specification is available under a Creative Commons Zero v1.0 Universal license. Additional instructions and software tools are hyper-referenced on this resource to support the adoption and use of DUC.
